# High-intensity lipid-lowering regimens in patients with stable coronary artery disease: the intriguing question of all-cause mortality

**DOI:** 10.1093/ehjcvp/pvz049

**Published:** 2019-09-27

**Authors:** Nicolas Danchin, Tabassome Simon

**Affiliations:** 1 Department of Cardiology, Hôpital Européen Georges Pompidou, AP-HP, 20 rue Leblanc, 75015 Paris, France; 2 Université Paris Descartes, 20 rue Leblanc, 75015 Paris, France; 3 FACT (French Alliance for Cardiovascular clinical Trials), 20 rue Leblanc, 75015 Paris, France; 4 Department of Pharmacology, Hôpital St Antoine, AP-HP, 27 Rue Chaligny, 75012 Paris, France; 5 Sorbonne Université, 27 Rue Chaligny, 75012 Paris, France

Statins at conventional doses have demonstrated the linear relationship between reduction in LDL-c and reduction in cardiovascular (CV) events; they increase life expectancy in primary and secondary prevention. Following the seminal trials, new trials have shown that high-intensity lipid-lowering treatment (LLT) could further reduce CV events, compared with conventional-dose statins. In stable coronary artery disease (CAD), however, high-intensity LLT, though decreasing potentially lethal CV events [acute myocardial infarction (AMI) and stroke] does not reduce mortality, compared with conventional-dose statins.

## High-intensity lipid-lowering treatment and risk of cardiovascular events

In stable CAD, high-dose LLT reduces CV endpoints vs. conventional-dose statins.^1–3^ Consequently, guidelines recommend targeting LDL-c levels <55 mg/dL^4^ or the routine use of high-dose statins^5^ (Grade IA), so that all CAD patients should receive lifelong high-intensity LLT.

Further trials tested whether improving LDL-c control by additional lipid-lowering agents on top of statins would improve clinical outcomes. IMPROVE-IT^6^ assessed ezetimibe in patients after acute coronary syndromes (ACS) followed for 7 years. FOURIER evaluated evolocumab, a PCSK9 inhibitor, on top of high-dose statins in stable patients.^7^ Both trials showed a reduction in combined CV endpoints compared with optimal statin therapy alone, bringing further evidence of the link between LDL-c and atherosclerotic disease.

## Conventional doses, high-intensity lipid-lowering treatment, and all-cause mortality

Compared with placebo, conventional-dose statins in stable CAD increase life expectancy [19% mortality reduction; relative risk (RR) 0.81, 95% confidence interval (CI) 0.75–0.88].^8^ Likewise, in primary prevention, a 14% mortality reduction observed (RR 0.86, 95% CI 0.79–0.94).^9^

In contrast, high-intensity LLT compared with conventional-dose statins in stable CAD has no effect on; none of the trials showed any mortality reduction,^1–3^^,^^7^ except an open-label, prematurely-stopped trial, with a high proportion of lost to follow-up.^10^ In trials testing 80 mg atorvastatin or simvastatin vs. lower doses, the hazard ratio (HR) for mortality stayed around 1.00 trial after trial, and remained such in IMPROVE-IT^6^ (post-ACS period followed by stabilized period) and FOURIER.^7^

**Figure 1:**
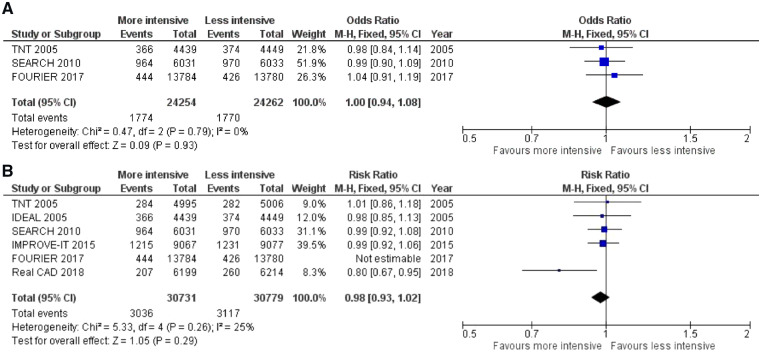
All-cause mortality in the larger trials comparing high-intensity vs. conventional lipid lowering in stable coronary artery disease patients. (*A*) Double-blind trials in stable patients. (*B*) Also including the open-label IDEAL and REAL-CAD trials and the long-term post-ACS IMPROVE-IT trial.

Therefore, despite dozens of thousands stable patients included, high-intensity LLT fails to document improved survival (*Figure 1*), although it reduces potentially lethal CV events (AMI risk ratio 0.82, 0.78–0.87 or stroke risk ratio 0.82, 0.76–0.89).

**Figure 2:**
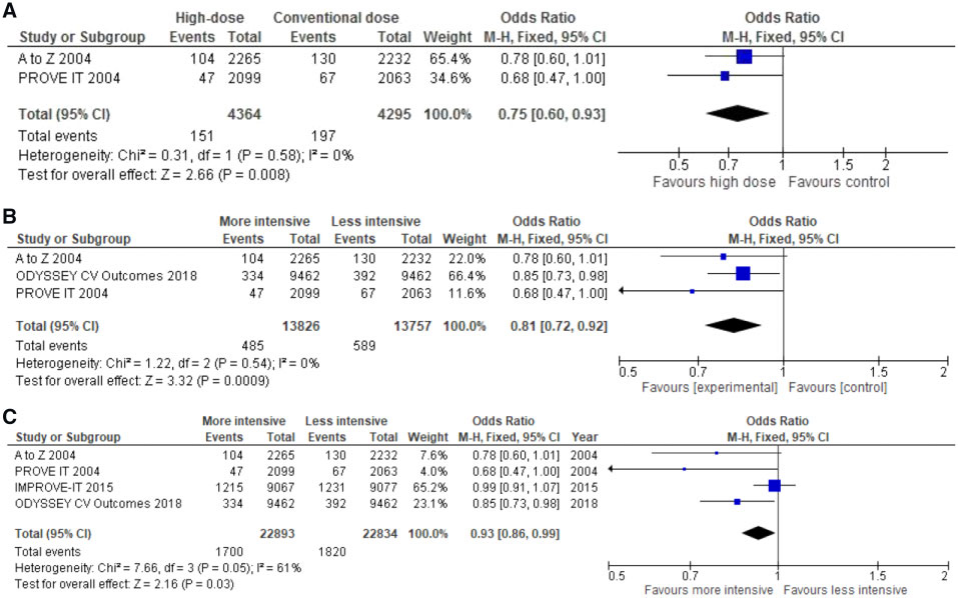
All-cause mortality in post-acute coronary syndrome trials. (*A*) High-dose statin trials with 2 years of follow-up. (*B*) Trials with high-dose statins or PCSK9-inhibition and with short duration of follow-up. (*C*) All trials with high-intensity lipid-lowering therapy, including the IMPROVE-IT trial (acute coronary syndrome with 6-year follow-up).

In post-ACS patients, however, mortality was numerically lower in the high-dose arms of the statin trials,^11^^,^^12^ as with alirocumab on top of statins.^13^ Together, these trials show mortality reduction, particularly those focusing on the early post-ACS period (*Figure 2*).

## Potential explanations for the lack of effect of high-intensity lipid-lowering treatment on mortality in stable patients

Lack of power, short follow-up, low rates of death, or competing mortality have been proposed as potential explanations.

- Admittedly, none of the individual trials were powered for mortality. The trial meta-analysis, including 76 661 patients has the power to document a reduction in mortality. In fact, mortality reduction was documented in much smaller populations when comparing conventional-dose statins vs. control in primary prevention trials. Lack of power is therefore not a plausible explanation.
- Apart from FOURIER,^7^ the duration of all the other outcome trials in stable patients was ≥5 years, enough for documenting treatment efficacy.
- It has been suggested that mortality has become so low, and the patients so well treated, that it is now virtually impossible to reduce mortality further. In the high-intensity LLT trials, mortality rates in the control groups were 1.14–2.55 per 100 patient-years, to compare with 1.81–2.94 in the first statin secondary prevention trials and 0.3–1.5 in primary prevention trials. Mortality rates in the high-intensity trials are thus between those in the primary prevention and conventional-dose secondary prevention trials, both of which documented a significant reduction in mortality. Likewise, annual death rates were similar in the ODYSSEY CV outcomes and FOURIER trials (1.46 and 1.45 per 100 patient-years); mortality was reduced in the post-ACS trial, but numerically higher in the trial in stable patients.^7^^,^^13^
- Competing mortality is an important issue. The greater longevity resulting from reduced CV mortality will ultimately result in increased non-CV death. But mortality from other causes should occur at a later stage, so that all-cause mortality should be lower at a given timepoint (e.g. 5 years).

## Perspectives

High-intensity LLT reduces myocardial infarction (MI) or strokes, potentially lethal complications either acutely or subsequently; by reducing MI/stroke, it might reduce non-CV mortality as well (a patient surviving an MI/stroke is less likely to survive, for instance, severe pneumonia or any non-CV surgery). Therefore, the neutral effect on mortality strongly suggests that the CV deaths spared by reducing MI/stroke are counterbalanced by other deaths, of unknown origin so far. As the mortality equipoise is around 5 years, prolonged high-intensity LLT beyond that timeframe might lead to more deaths induced than lives saved (admittedly, the reverse could also be true).

These data also raise the issue of personalized and adaptive therapy; most probably, some patients would benefit from more potent LLT while others might be harmed. Moreover, as high-intensity regimens reduce mortality in the early post-ACS phase but not in stable patients, it is possible that the same doses or types of treatments should not be used forever in CAD patients (e.g. it might be wiser to use high-intensity therapy for a limited period following an ACS, then reverting to conventional doses in most patients).

Moreover, individual data from high-intensity LLT trials should be made available for independent analyses of potential benefit/harm, of more intensive treatment, related to treatment duration and patient subsets. Monitoring observational cohorts would have added value.

To date, the only landmark analysis of mortality comes from FOURIER,^7^ with numerically lower CV mortality with evolocumab during the first year (HR 0.96; 0.74–1.25), but higher thereafter (HR 1.12, 0.88–1.42). To the best of our knowledge, all-cause mortality has not been analysed by subgroups or follow-up duration in stable patients.

Currently, whereas treat-to-target or doses have been extensively debated, the relevance of lifetime high-dose LLT is seldom addressed. At this stage, it might be more reasonable for guidelines to simply consider lifelong use of high-intensity therapies, rather than recommending them as mandatory forever in all stable CAD patients.

**Conflict of interest:** Professor Danchin and Simon have received fees for conferences or counselling and/or research grants from most companies marketing statins, from MSD which markets ezetimibe, from AMGEN (evolocumab) and from Sanofi (alirocumab). Moreover, Dr Danchin, as the national coordinator for France, and Simon, as co-coordinator of FACT academic research organization, have been involved in the ODYSSEY cardiovascular outcomes trial with alirocumab.
